# Outcomes of Total Laparoscopic Hysterectomy: A Single-Surgeon Experience of Initial 50 Cases

**DOI:** 10.7759/cureus.12644

**Published:** 2021-01-12

**Authors:** Sana Ashfaq, Mubashra Samina, Maria Jabeen, Shaheen Zafar

**Affiliations:** 1 Obstetrics and Gynaecology, Atia General Hospital/Koohi Goth Hospital, Research and Training Center, Karachi, PAK; 2 Obstetrics and Gynaecology, Atia General Hospital, Karachi, PAK; 3 Obstetrics and Gynaecology, Liaqat National Hospital, Karachi, PAK

**Keywords:** benign diseases, complications, total laparoscopic hysterectomy

## Abstract

Introduction

In this study, we reported a single surgeon experience of total laparoscopic hysterectomy (TLH) in terms of intraoperative and early postoperative outcomes and complications. In addition, we compared our results with published literature for a reevaluation of complications and outcomes.

Material and methods

This present prospective study was conducted on 50 patients who underwent TLH due to benign causes. Patients diagnosed with abnormal uterine bleeding (AUB), uterine fibroids, and post-menopausal bleeding (PMB) were included in this analysis. Patients were discharged after 24 hours of surgery if there were considered fit for discharge. The patients' age, co-morbidities, size of the uterus, additional procedure along with TLH, and postoperative complications were collected and analyzed. The follow-up period was three months, done on the tenth day after surgery, the thirtieth day, and then at three months.

Results

The mean age of our patients was 46.42±5.01 years. The major indication of hysterectomy was fibroids diagnosed in 27 (54.0%) patients and AUB in 18 (36.0%) patients. Out of 50, 10 (20.0%) patients had a previous cesarean section, and 4 (8.0%) had a bilateral tubal ligation (BTL). Mean surgery duration was 124.26±44.74 minutes. Mean hospital stay was 2.18±0.39 days. Total complications occurred in five (10.0%) patients, ureteric injury in one (2.0%) patient, port-site infections in 2 (4.0%), and vault infections in 2 (4.0%) patients.

Conclusion

TLH is a safe procedure and can be performed with minimal complications in patients with benign uterine etiology.

## Introduction

After a cesarean section, total laparoscopic hysterectomy (TLH) is the second most common procedure in gynecology departments [1]. Abnormal uterine bleeding (AUB), pelvic organ prolapse, leiomyoma, and cancer are routine indications for TLH [2]. TLH is the preferred approach over other procedures, such as laparotomy, because of fewer complications and higher access rates [3]. Vaginal hysterectomy is shown to have fewer complications than the laparoscopic approach but its access is limited in many procedures [4-5]. Vaginal hysterectomy is more difficult to perform in older women with prolapse, especially when the procedure involves adnexal surgery because pelvic access is difficult using the vaginal approach in these patients [6]. Therefore, TLH has now become the preferred approach for benign conditions.

However, despite these benefits and acceptable clinical outcomes, TLH has not gained widespread importance. Even in the USA, the number of TLHs procedures has decreased instead of increasing. In the USA, the number of TLHs performed was 15.5% in 2006, and it reduced to 8.6% in 2010 [2]. This may be due to the increase in robotic hysterectomy in the USA [2]. The other reasons are that the laparoscopic approach requires a steep learning curve and is challenging in obese patients [7]. Furthermore, there is also a risk of injury to the urinary tract during the procedure, and the operative time is prolonged as compared to other approaches [5]. Due to these reasons even in Europe, only a minor proportion of total procedures is done laparoscopically [6,8].

With the passage of time, gynecology consultants have gained more experience, therefore, the operative time and complications of TLH have decreased. In the present study, we reported a single surgeon experience of TLH in terms of intraoperative and early postoperative outcomes and complications. In addition, we compared our results with published literature for the reevaluation of complications and outcomes after TLH.

## Materials and methods

This present prospective study was conducted on 50 patients who underwent TLH due to benign causes. Patients diagnosed with AUB, uterine fibroids, and postmenopausal bleeding (PMB) were included in this analysis while cases with malignancy were excluded. The study duration was from August 2018 to December 2019. Before surgery, all patients were informed about TLH and its potential benefits and written consent was taken for participation in the study.

Routine investigations, including complete clinical history, complete blood profile, ultrasonography, and biopsy studies, were done to rule out any malignant pathology.

A single consultant performed all laparoscopic procedures. All procedures were done by a consultant gynecologist. The procedure was done after inducing general anesthesia by placing the patient in the lithotomy position, with legs spread apart with small flexion at the hip joint (to obtain lateral movements during surgery). The following steps were followed during hysterectomy: coagulation of upper pedicles, opening of vesicoperitoneal folds, skeletonization of uterine vessels, coagulation of vessels, colpotomy, followed by specimen retrieval vaginally. For keeping the pneumoperitonimum, vaginal occlusion was done. The vaginal vault was closed by continuous suturing by laparoscopy.

After surgery, patients were discharged after 24 hours of surgery if they were considered as fit for discharge. The patients' age, co-morbidities, size of the uterus, additional procedure along with TLH, and postoperative complications were collected and analyzed. The follow-up period was three months, done on the tenth day after surgery, the thirtieth day, and then at three months.

For data analysis, we used the Statistical Package for the Social Sciences (SPSS) v23 (IBM Corp., Armonk NY). The analysis of variance (ANOVA) test was applied to compare mean operative time and hospital stay in patients with isolated TLH and those with concomitant procedures by considering p-value ≤0.05 as significant statistically.

## Results

The mean age of patients was 46.42±5.01 years. Hepatitis was the most common morbidity presented in nine (18%) patients; hypertension and diabetes mellitus presented in five (10%) and five (10%) patients, respectively. The major indication of hysterectomy was fibroids diagnosed in 27 (54%) patients and AUB in 18 (36%). The full etiologic spectrum is given in Table 1. Regarding previous history, 10 (20%) patients had a history of C-section, four (8%) had a bilateral tubal ligation (BTL), one (2%) had laparotomy, and one (2%) had an appendectomy. Isolated TLH was done in 31 (62%) patients, TLH+BSO in 17 (24%) patients, TLH+RSO in one (2%) patient, and TLH+LSO in one (2%) patients. The mean surgery duration was 124.26±44.74 minutes (Table 1). The mean surgery time was not significantly different in TLH alone versus those with the concomitant procedure (Table 1).

**Table 1:** Baseline study variables TLH, total laparoscopic hysterectomy; AUB, abnormal uterine bleeding; PMB, post-menopausal bleeding; BSO, bilateral salpingo-oophorectomy; RSO, right salpingo-oophorectomy; LSO, left salpingo-oophorectomy

Age	46.42±5.01
Comorbidities	
Hepatitis B	1 (2.0%)
Hepatitis C	9 (18.0%)
Hypertension	5 (10.0%)
Diabetes Mellitus	5 (10.0%)
Indications of TLH	
AUB	18 (36.0%)
Uterine Fibroids	27 (54.0%)
PMB	01 (2.0%)
Adenomyosis	03 (6.0%)
Cerebral Palsy (CP) Child	01 (2.0%)
Size of Uterus	13.16±2.39
Previous Surgical History	
C-Section	10 (20.0%)
Bilateral Tubal Ligation (BTL)	4 (8.0%)
Laparotomy	1 (2.0%)
Laparoscopic Appendectomy	1 (2.0%)
Type of Surgical Procedure	
TLH	31 (62.0%)
TLH+BSO	17 (24.0%)
TLH+RSO	01 (2.0%)
TLH+LSO	01 (2.0%)
Duration of Surgery (mins)	124.26±44.74

Total complications occurred in five (10%) patients; there were no major complications and only minor complications were diagnosed. Out of five, a ureteric injury occurred in one (2%) patient, port-site infections were diagnosed in two (4%), and vault infections in two (4%) patients. The mean hospital stay was 2.18±0.39 days (Table 2).

**Table 2:** Procedural complications of TLH TLH, total laparoscopic hysterectomy

Mean Hospital Stay (days)	2.18±0.39
Need of Laparotomy	0
Paralytic Ileus	0
Bladder Injury	0
Ureteric Injury	1 (2.0%) (Rt. Ureter)
Visceral Injury	0
Mortality	0
Need for Re-operation	0
Port-Site Infections	2 (4.0%)
Vaginal Bleeding	0
Vault Infections	2 (4.0%)
Vaginal Evisceration	0
Re-suturing of Vault	0
Vault Hematoma	0
Port-Site Hernia	0
Blood Transfusion	0

The mean hospital stay was not influenced by the type of surgical procedure (Table 3).

**Table 3:** Comparison of mean operative time and hospital stay in patients with TLH alone and with concomitant procedures. TLH, total laparoscopic hysterectomy; BSO, bilateral salpingo-oophorectomy; RSO, right salpingo-oophorectomy; LSO, left salpingo-oophorectomy

	TLH Alone	TLH+BSO	TLH+RSO	TLH+LSO	P-value
Operative Time	129.74±48.49	111.58±33.94	194.0	100	0.21
Hospital Stay	2.22±0.42	2.11±0.33	2.0	2.0	0.74

## Discussion

Laparoscopic procedures are routine practice in gynecology departments for nearly the last 30 years [8-9]. In some countries like Pakistan, the use of LH has increased over some decades. LH can be performed using several techniques. In 2003, Reich et al. defined the terminology of LH and defined TLH as a procedure in which the whole procedure is performed through a laparoscope except for the removal of the uterus [8].

In the present study, we reported a single surgeon’s experience of intraoperative and postoperative outcomes of TLH in benign diseases. In the present study, the mean age was 46.42±5.01 years. Mereu et al. reported a mean age of 49.6 ± 6.5 years among patients who underwent TLH due to benign diseases [10]. Shim et al. also reported a mean age of 48 years [11].

The most common indication of TLH in our study was uterine fibroids presented in 54% cases and AUB in 36% cases. Agarwal et al. reported fibroids in 51.2% of patients, AUB in 28%, adenomyosis in 19.2%, and hyperplasia in 1.6% of patients who underwent TLH. While for TAH, they reported fibroids in 48%, AUB in 30.4%, adenomyosis in 20%, and hyperplasia in 5.6% of patients [3].

In our study, the mean operative time was 124.26±44.74 minutes. Studies have reported differences in mean operative time. Kim et al. reported a mean operative’ time of 149.3 ± 59 minutes [12]. Ng et al. reported a mean time of 136 minutes [13]. Their operative time was longer than our study. While studies by Wallwiener et al. and Boosz et al. reported a shorter operative time - 103 ± 36 minutes and 104 ± 44 minutes, respectively [14-15].

Regarding complications, we did not encounter any incidence of conversion to laparotomy and hospital mortality in our case series. There were only three types of complications that we encountered: ureteric injury in 2%, port-site infections in 4%, and vault infections in 4% of patients with an overall complication rate of 10%.

A study by Mereu et al. reported bladder injury in 0.3%, and blood transfusion in 0.3% of patients, port-site hernia in 0.3%, wound infections in 1.1%, vaginal cuff dehiscence in 0.3%, and vaginal cuff bleeding in 0.8% after TLH. The overall complication rate was 4.4%. They did not report an incidence of conversion to laparotomy or any hospital mortality [10].

A study by Agarwal et al. reported that years of experience of TLH has a significant influence on the complication rate of TLH. They reported complications of 14% in the first year of TLH, and it reduced to only 4.3% after three years of experience. The rate of conversion to laparotomy in the first year was 9.6%, and it decreased significantly within four years [3].

A study by Twijnstra et al. reported a need for laparotomy in 4.8% of patients, bladder injury in 1.4%, ureteral injury in 0.4%, and bowel injury in 0.5% of patients [16].

The mean hospital stay in our study was 2.18±0.39 days. Our length of stay was shorter when compared to Kim et al. and Wallwiener et al., who reported a hospital stay of 5.5±2.0 days and 4.9±2.8 days, respectively [12,14]. This was comparable to the studies by Mereu et al., who reported a hospital stay of 2.6±1.1 days [10].

The major limitation of this study is its non-randomized nature and small sample size because of the low volume of patient flow for hysterectomies in our hospital. However, we have compared our results with international literature, which has enabled us to verify the outcomes of TLH in our patients.

## Conclusions

TLH is a safe procedure and can be performed with a minimal number of complications. Initial studies reported a higher complication rate, but the latest studies, including the present one, have found a very low rate of procedural complications. So this procedure can be adopted as the preferred approach in patients planned for hysterectomy due to benign diseases.
